# Real-time aortic pulse wave velocity measurement during exercise stress testing

**DOI:** 10.1186/s12968-015-0191-4

**Published:** 2015-10-05

**Authors:** Paul A. Roberts, Brett R. Cowan, Yingmin Liu, Aaron C. W. Lin, Poul M. F. Nielsen, Andrew J. Taberner, Ralph A. H. Stewart, Hoi Ieng Lam, Alistair A. Young

**Affiliations:** Auckland Bioengineering Institute, University of Auckland, Auckland, New Zealand; Department of Anatomy with Radiology, Faculty of Medical and Health Sciences, University of Auckland, 85 Park Road, Auckland, 1142 New Zealand; Greenlane Cardiovascular Unit, Auckland City Hospital, Auckland, New Zealand; Department of Engineering Science, University of Auckland, Auckland, New Zealand

**Keywords:** Aortic stiffness, Pulse wave velocity, Exercise stress test, Real time imaging

## Abstract

**Background:**

Pulse wave velocity (PWV), a measure of arterial stiffness, has been demonstrated to be an independent predictor of adverse cardiovascular outcomes. This can be derived non-invasively using cardiovascular magnetic resonance (CMR). Changes in PWV during exercise may reveal further information on vascular pathology. However, most known CMR methods for quantifying PWV are currently unsuitable for exercise stress testing.

**Methods:**

A velocity-sensitive real-time acquisition and evaluation (RACE) pulse sequence was adapted to provide interleaved acquisition of two locations in the descending aorta (at the level of the pulmonary artery bifurcation and above the renal arteries) at 7.8 ms temporal resolution. An automated method was used to calculate the foot-to-foot transit time of the velocity pulse wave. The RACE method was validated against a standard gated phase contrast (STD) method in flexible tube phantoms using a pulsatile flow pump. The method was applied in 50 healthy volunteers (28 males) aged 22–75 years using a MR-compatible cycle ergometer to achieve moderate work rate (38 ± 22 W, with a 31 ± 12 bpm increase in heart rate) in the supine position. Central pulse pressures were estimated using a MR-compatible brachial device. Scan-rescan reproducibility was evaluated in nine volunteers.

**Results:**

Phantom PWV was 22 m/s (STD) vs. 26 ± 5 m/s (RACE) for a butyl rubber tube, and 5.5 vs. 6.1 ± 0.3 m/s for a latex rubber tube. In healthy volunteers PWV increased with age at both rest (R^2^ = 0.31 *p* < 0.001) and exercise (R^2^ = 0.40, *p* < 0.001). PWV was significantly increased at exercise relative to rest (0.71 ± 2.2 m/s, *p* = 0.04). Scan-rescan reproducibility at rest was −0.21 ± 0.68 m/s (*n* = 9).

**Conclusions:**

This study demonstrates the validity of CMR in the evaluation of PWV during exercise in healthy subjects. The results support the feasibility of using this method in evaluating of patients with systemic aortic disease.

## Background

Stiffness of the aorta is an important determinant of cardiovascular function [1, 2] due to its direct effect on systolic and diastolic blood pressure. Aortic pulse wave velocity (PWV) is a surrogate measure of aortic stiffness which has been shown to independently predict adverse cardiovascular events and mortality [3–5]. Estimation of PWV by cardiovascular magnetic resonance (CMR) has been demonstrated to give good reproducibility in scan-rescan evaluations, with measurements superior to echocardiography [6]. Variations in PWV with age and vascular pathologies have also been reported [2, 7, 8]. However, little is known on how aortic PWV changes with exercise.

Although cardiac function at rest provides important information, the power to detect dynamic cardiovascular dysfunction is greatly enhanced by stress testing [9, 10]. Pharmacological stress tests with agents such as adenosine and dobutamine have commonly been employed in conjunction with CMR examinations [11]. However, exercise provides a physiological challenge to the entire cardiovascular system [12], and is generally regarded as superior to pharmacological stress testing. This is particularly important when evaluating arterial response to states of increased cardiac output. Since the aortic wall exhibits nonlinear mechanical properties [13], aortic stiffness during exercise is expected to change due to pressure loading [14, 15]. PWV measurement during exercise stress testing may, therefore, provide additional useful information on disease status beyond that seen in the resting state.

The aim of this study was to examine and validate a method of quantifying aortic PWV during exercise using CMR. The method utilized a real-time velocity sensitive acquisition, automated quantification of PWV, and validation in phantoms. Quantification of scan-rescan reproducibility was also assessed.

## Methods

### Ergometer

**Fig. 1:**
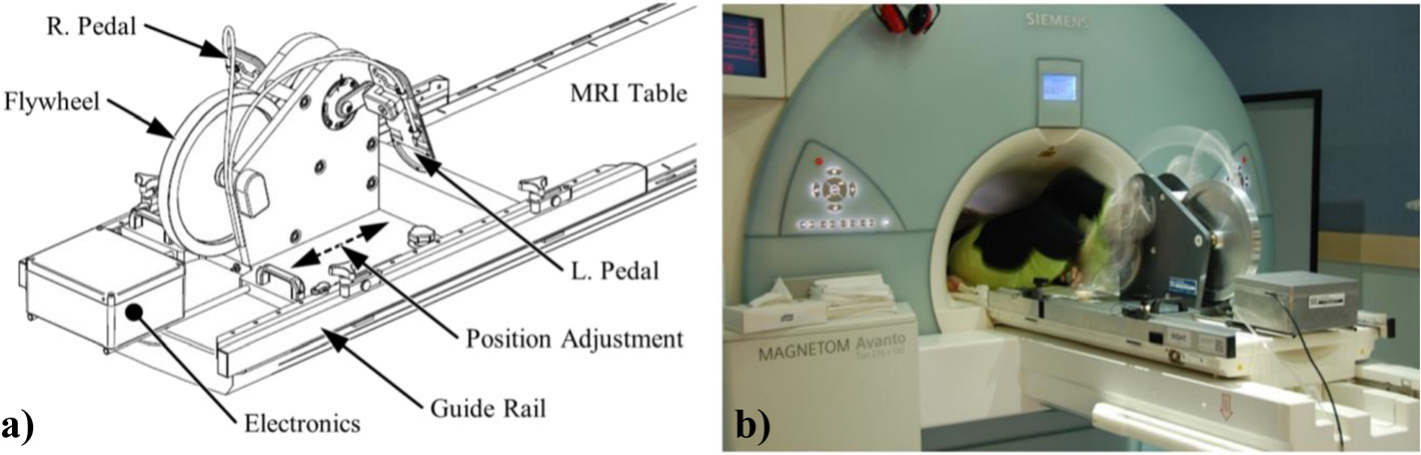
Custom-built CMR compatible ergometer. **a** Schematic showing adjustable position to accommodate different body sizes. **b** Volunteer cycling in the scan position

A custom-built CMR-compatible cycle ergometer [16] was adapted for this study (Fig. 1). The ergometer comprised a set of pedals mounted on 60 mm radius cranks coupled to a hub driving an aluminium flywheel with 6:1 ratio using toothed sprockets and belts. Force transducers (LRF350, FUTEK Advanced Sensor Technology, Inc., Irvine, CA, USA) measured the load applied to each pedal and an optical encoder (HEDS-5540, Avago Technologies, San José, CA, USA) measured flywheel rotational speed. Sensor signals were transmitted through short shielded cables to a battery-powered conditioning and USB digitization device (USB-6210, National Instruments, Austin, TX, USA) mounted on the ergometer, and then transmitted via a USB fibre-optic link (USB Rover 200, Icron Technologies Corporation, BC, Canada) through a wave guide to a computer in the MRI control room. Custom software (LabVIEW 2009, National Instruments, Austin, TX, USA) was written to calculate and display work rate, and enable ergometer resistance adjustment, as follows.

A pneumatic actuator (CJPB10-15H6, SMC Corporation, Tokyo, Japan) pressed a 21 mm diameter braking felt pad to the outer rim of the flywheel. The actuator’s air supply pressure determined the force on the pad and hence resistance. Compressed air from a 2.7 L dive cylinder located in the control room was regulated to 1000 kPa by a first stage dive regulator (R1, Atlantis Dive, Auckland, NZ). This fed a 0–500 kPa electronic pressure regulator (ITV0031-2CL, SMC Corporation, Tokyo, Japan) adjusted under software control using the regulator’s 0–5 V_DC_ analogue input via a USB interface (USB-6008, National Instruments, Austin, TX, USA). Polyurethane tubing connected the air supply to the scan room through a wave-guide thereby delivering the regulated air to the actuator.

### Subjects

The study was approved by the Multi-Region Ethics Committee of the New Zealand Health and Disability Ethics Committees, and written informed consent was obtained from all participants. Fifty healthy volunteers (28 male, aged 22–75 years) completed rest and exercise protocols. Strenuous exercise and caffeine were avoided for 24 h, and food and alcohol were avoided for a minimum of 3 h prior to CMR examination. Target work rate was set to raise heart rate by approximately 30 bpm above resting baseline. Exclusion criteria included pregnancy, contraindication to CMR, abnormal ECG or atrial fibrillation, physical limitations preventing cycle exercise, known cardiovascular disease, current smoker or ceased smoking within 6 months, and treated or untreated hypertension >140/90 mmHg (>18.7/12.0 kPa) [17].

### Study protocol

All studies were performed using a 1.5 T MRI scanner (Avanto, Siemens AG Healthcare Sector, Erlangen, Germany). The ergometer was fixed to the scanner table with participants in a supine position (Fig. 1b), and adjusted to position the heart to within 100 mm of iso-centre while avoiding knee contact with the scanner while pedaling.

**Fig. 2:**
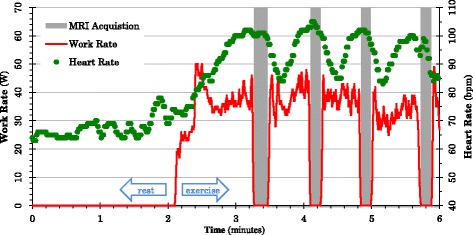
Work rate (*red*) and heart rate (*green*) during the rest and exercise phases of the protocol. *Grey bands* indicate breath-hold acquisitions when cycling is ceased

The imaging protocol included standard scouts to locate the axes of the heart, cine ventricular function, aortic PWV, and blood pressure with pulse wave analysis at rest and immediately after exercise. For exercise data acquisitions, participants cycled at the target work rate until their heart rate stabilized, and image acquisition was performed immediately after cessation of cycling with a short breath-hold at expiration (Fig. 2). After each acquisition, participants were instructed to resume cycling at the target work rate.

Six short axis slices, equally spaced from base to apex, and two long axis slices (four chamber and two chamber views) were acquired using a 3× accelerated balanced steady state free precession sequence [18]. Typical parameters were: Base resolution 128 pixels, field of view (FOV) 300 mm, 82 % rectangular FOV, phase resolution 100 %, slice thickness 6 mm, 6–15 views per segment, TR 2.6 ms, TE 1.1 ms, and flip angle 80°. Temporal resolution was 16–40 ms, dependent on heart rate, enabling acquisition of two slices per breath-hold of 8–10 s duration at rest, and 5–7 s duration after exercise.

CMR aortic PWV measurement was performed using a real-time acquisition and evaluation (RACE) sequence, originally described by Bock et al. [19]. The RACE PWV method enabled flow sensitive 1D projection imaging of two independent slices at 7.8 ms temporal resolution per slice with a 4 s breath-hold. Briefly, a gradient-echo sequence was modified to make the signal phase dependent on velocity using a through-slice velocity-encoding gradient. A 1D projection of the 2D slice was produced perpendicular to the readout direction by omitting the phase-encoding gradient. By orientating the slice perpendicular to the vessel of interest and ensuring no other vessels were in the same projected voxel, a velocity signal could be obtained as a combination of stationary and moving spins. Unlike standard phase-contrast flow sequences, there was no acquisition of a flow-compensated signal, which further contributed to improving the temporal resolution.

**Fig. 3:**
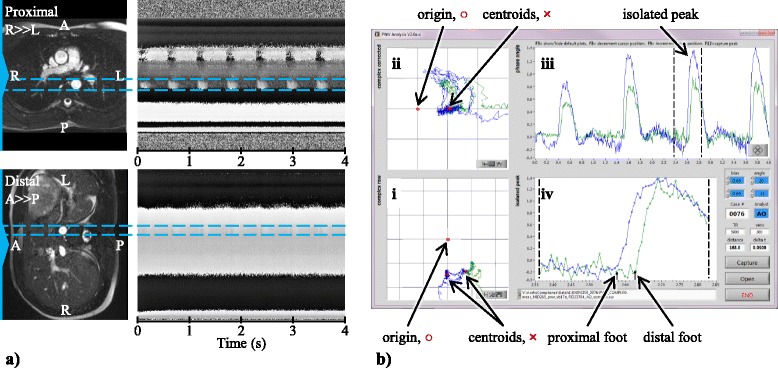
MRI PWV analysis. **a** Region of interest selection guided by anatomical images (*left*) and corresponding RACE phase image (*right*). **b** Correction for stationary tissue signal in the complex domain, with proximal (superior) slice signal in the vessel region shown in *blue* and distal (inferior) signal in *green*. **b**-*i* Raw data plotted in the complex plane, showing centroids marking slow flow signal. **b**-*ii* Complex signal relative to the reference static tissue point. **b**-*iii* Phase waveforms relative to the reference point. **b**-*iv* Estimation of foot transit time

The sequence parameters were velocity encoding (VENC) 250 cm/s, TR 3.9 ms, TE 1.9 ms, flip angle 15 °, image matrix 512 (pixels) × 512 (time points), bandwidth 673 Hz/pixel, slice thickness 5 mm, and FOV 300 mm. Signals were acquired at two axial slices located at the level of the pulmonary bifurcation and above the renal and superior mesenteric arteries in alternating TRs, achieving 7.8 ms temporal resolution for each slice. Data were acquired for 512 lines for each slice, giving a total acquisition time of 4 s. The phase-encode direction was set to be right-left for the superior slice, and anterior-posterior for the inferior slice (Fig. 3), in order to avoid overlap of other major vessels in the projection direction. Two acquisitions were performed during separate breath-holds and the results concatenated.

Blood pressures were acquired at rest and immediately after exercise using an automated sphygmomanometer (CardioScope I, Pulsecor Ltd, Auckland, NZ), which also estimated the central aortic pressure from analysis of low-frequency suprasystolic waveforms at the occluded brachial artery. This device has previously been validated against invasive pressure recordings [20].

### Analysis

All images were randomized so that analysts were blinded to participants, rest and exercise. Left ventricular mass and volume were determined using guide-point modeling [21]. Briefly, a spatio-temporal finite element model was interactively customized to all short and long axis slices and frames simultaneously. Breath-hold mis-registration was corrected manually with in-plane translations. Mass was calculated by numerical integration of the model and averaged over all frames. The time-varying position and angulation of the mitral valve plane provided the basal cut-off for the calculation of mass and volume. Previous studies have shown that this method gives accurate estimation of mass and volume with a reduced number of non-contiguous short axis slices in humans [22] and mice [23].

Figure 3 shows the PWV analysis method. The vessel region of interest was selected to encompass the pulsatile flow signal in the RACE phase image (Fig. 3a) matching the location of the cross-section of the descending aorta in an anatomical scout image acquired with the same imaging parameters (FOV and slice position) as the RACE acquisition. The complex velocity-sensitive signal was averaged across the region of interest for all time points. The stationary tissue signal component was identified and subtracted using a two-step procedure. Firstly, the data were plotted for all time points on the complex plane, and the centroid of the signal data during low or zero flow was calculated by successively eliminating 66 % of the points furthest from the centroid. Secondly, a complex vector representing static tissue was subtracted from each data point. The position of this reference signal was designed to provide a robust measure of upstroke during the pulsatile flow, approximately in the center of the signal arc of flowing spins which have a flow-dependent magnitude [19]. This point was defined to have the same phase as the centroid but was offset from the centroid towards the origin by a distance of half the magnitude of the peak pulsatile flow signal relative to the centroid (Fig. 3b). The phase of the complex difference was then plotted against time and the PWV determined automatically by the early systolic fit method [24, 25] (Fig. 3b). Following [24, 25], the upstroke between 20 and 40 % of the peak value was fitted by a least squares line and intersected with the baseline, which was defined to be the minimum of the 10 points prior to the upstroke, to find the foot of each heart beat. The foot-to-foot time difference (Δt) between proximal and distal slices was collected for all heart beats and the median time difference was used in the following equation to calculate PWV:

1 $$ PWV=\frac{\varDelta d}{\varDelta t} $$

where Δ*d* is the distance between slices.

### Pulsatile flow phantom

**Fig. 4:**
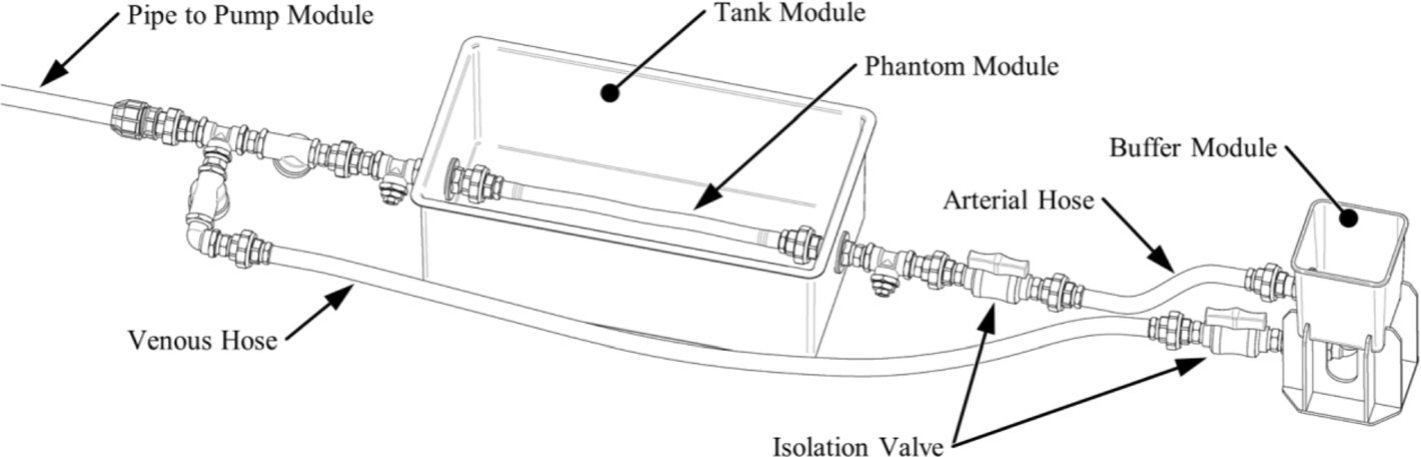
Flow circuit for phantom experiments

PWV from the CMR RACE sequence was compared to PWV estimates obtained using a standard 2D gated phase contrast (PC) flow sequence (Siemens AG Healthcare Sector, Erlangen, Germany) in a pulsatile flow phantom. Two phantoms of different compliance were tested. A 19 mm diameter latex rubber tube and a stiffer 21 mm butyl rubber tube were mounted in a tank within a volume of water. A linear motor (STA2510, Copley Motion Systems LLC, Essex, UK) under software control drove a piston to push water through a circuit of pipes and one-way valves, generating repetitive forward moving pulsatile flow through the compliant phantom section which returned through a separate hose (Fig. 4). Axial slices were acquired at ±150 mm from the iso-centre, during a 60 cycles per minute simulation with 30 and 50 mL stroke volumes for the latex and rubber phantoms, respectively. RACE imaging parameters were the same as described above. The standard 2D gated PC flow acquisition parameters were VENC 1.5 m/s, TR 9.6 ms, TE 2.0 ms, flip angle 30 °, bandwidth 554 Hz/pixel, slice thickness 5.5 mm, FOV 320 mm, and 69 % rectangular FOV with a 55 s acquisition time. Flow encoding was through-plane at the same axial slices (orthogonal to the phantom) as the RACE sequence.

### Scan-rescan reproducibility

RACE PWV repeatability was assessed in nine asymptomatic volunteers who underwent two evaluations at rest. These were performed consecutively to minimize diurnal variations and separated by removing volunteers from the scanner. Each evaluation consisted of localizers, and two sequential breath-hold PWV acquisitions. The median pulse transit times from the sequential data sets were used to estimate aortic PWV scan-rescan variability.

### Statistics

Rest and exercise data were tested with two tailed paired *t*-tests, and differences due to gender with independent *t*-tests. Significance level of 0.05 was assumed. The relationship between age and aortic PWV was tested using linear correlation and the interaction with exercise was tested using ANCOVA.

## Results

### Validation studies

**Fig. 5:**
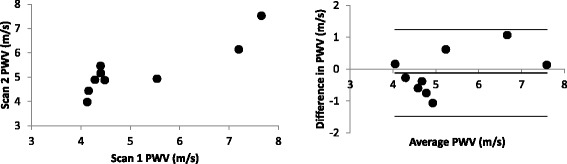
Scatterplot and Bland-Altman plot of scan-rescan reproducibility in aortic PWV (*n* = 9, rest)

PWV were 22 m/s (standard PC) vs. 26 ± 5 m/s (RACE) for the stiffer butyl rubber phantom, and 5.5 m/s (standard PC) vs. 6.1 ± 0.3 m/s (RACE) for the compliant latex rubber phantom. Over all phantom experiments, the standard deviation in transit times averaged 2 ms, approximately half the TR of the RACE sequence. Additional experiments over five different slice spacings ranging from 100 to 300 mm in the more compliant phantom resulted in approximately constant PWV averaging 5.2 ± 0.3 m/s. Scan-rescan reproducibility from 9 volunteers at rest was −0.21 ± 0.68 m/s (*p* = NS) (Fig. 5). Central mean arterial pressures were slightly lower in the second evaluation (−4 ± 3 mmHg, *p* < 0.01). Heart rate was similar in both evaluations (−3 ± 6 bpm, *p* = NS).

### Ventricular function

Demographics for the 50 asymptomatic volunteers are shown in Table 1. Changes in haemodynamic parameters from rest to exercise are presented in Table 2. Heart rate increase ranged from 12 to 75 bpm (average 31 ± 12 bpm) and work rate ranged from 13 to 109 W (average 38 ± 22 W). Changes in left ventricular functional parameters were all statistically significant (*p* < 0.01). Approximately 80 % of the cardiac output increase was due to heart rate increase, while the remainder was due to increased stroke volume. Mean arterial pressure increased by 5 ± 5 mmHg between rest and exercise (*p* < 0.001). Mean arterial pressure at rest was positively correlated with age at rest (R^2^ = 0.17, *p* = 0.003) and exercise (R^2^ = 0.11, *p* = 0.02); ANCOVA showed no significant interaction between exercise and age.

**Table 1:** Participant demographics (*n* = 50, 28 male)

Parameter	Mean ± std.dev.	Range
Age (years)	52.6 ± 15.0	22–75
Height (cm)	172.3 ± 9.2	154–193
Weight (kg)	74.8 ± 13.6	50–126
BMI (kg/m^2^)	25.1 ± 3.6	18.6–35.6
BSA (m^2^)	1.9 ± 0.2	1.5–2.6

**Table 2:** Changes from rest to moderate exercise

	Rest	Exercise	Change
Heart rate (bpm)	68 ± 12	99 ± 13	31 ± 12**
Work rate (W)	–	38 ± 22	–
SBP (mmHg)	120 ± 14	134 ± 16	14 ± 9**
DBP (mmHg)	74 ± 10	76 ± 9	2 ± 4**
MAP (mmHg)	87 ± 10	92 ± 10	5 ± 5**
PP (mmHg)	47 ± 8	59 ± 13	12 ± 9**
Central SBP (mmHg)	111 ± 14	123 ± 16	11 ± 9**
Central DBP (mmHg)	75 ± 10	78 ± 9	3 ± 4**
Central MAP (mmHg)	90 ± 12	97 ± 11	7 ± 6**
Central PP (mmHg)	37 ± 8	45 ± 12	8 ± 8**
Cardiac output (L/min^1^)	6.3 ± 1.4	10.2 ± 2.2	3.9 ± 1.9**
RPP (×10^3^ bpm·mmHg)	8.1 ± 1.7	13.3 ± 2.8	5.2 ± 2.5**
Cardiac output power (W)	1.22 ± 0.33	2.11 ± 0.62	0.87 ± 0.50**
End diastolic volume (mL)	146 ± 28	151 ± 31	5 ± 14*
End systolic volume (mL)	52 ± 14	47 ± 13	−5 ± 6**
Stroke volume (mL)	93 ± 16	104 ± 21	10 ± 13**
Ejection fraction (%)	64 ± 4	69 ± 4	5 ± 3**
LV mass (g)	125 ± 31	–	–
Augmentation index (%)	56 ± 29	40 ± 26	−15 ± 19**
PWV (m/s^1^)	5.5 ± 1.7	6.2 ± 2.1	0.7 ± 2.2*

*SBP* systolic blood pressure, *DBP* diastolic blood pressure, *MAP* mean arterial blood pressure, *PP* pulse pressure, *RPP* rate pressure product, *PWV* pulse wave velocity

*p < 0.05, **p < 0.001

### PWV

**Fig. 6:**
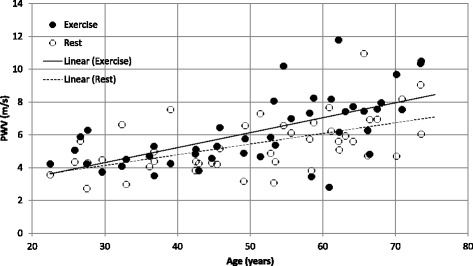
Aortic PWV versus age, at rest (*open circles*) and exercise (*solid circles*)

PWV could not be determined at either rest or exercise in 6 participants, due to noisy waveforms and inability of the algorithm to automatically calculate transit time. The average distance between the proximal and distal slices was 156 ± 17 mm. PWV was positively correlated with age, at both rest and during exercise (Fig. 6, R^2^ = 0.31 for rest and 0.40 for exercise, *p* < 0.001). At rest, PWV was 4.2 ± 1.0 m/s for participants aged 20–30 year (*n* = 6), increasing to 7.0 ± 2.0 m/s for participants aged 70–80 year (*n* = 4). PWV was significantly increased at exercise relative to rest (*p* = 0.04). ANCOVA, with age as a covariate, found no significant interaction between exercise and age.

## Discussion

To evaluate PWV in conditions of elevated heart rate, a fast acquisition with high temporal resolution and short breath-hold duration is essential. Several methods have been proposed for CMR PWV measurement, including 4D flow [26], two slice cine flow [27], and flow-area in a single slice [28]. However, these methods have limited application in exercise testing due to long breath-hold duration. Patients typically cannot hold their breath for more than 5 s under exercise conditions. Several fast acquisition methods have been proposed previously, including cylindrical excitation 1D flow [29], 1D flow spectroscopy [30], cylindrical excitation tagging [31], multislice comb RF excitation and 1D readout [32], complex difference of velocity encoded projections [33], and flow-sensitive RACE with stationary tissue suppression [19]. The RACE method was chosen for the current study because it offered high temporal resolution without the need for a velocity compensated acquisition or complex excitation procedure, both of which reduce the effective temporal resolution.

Langham et al. [33] used a similar 1D projection method in a single slice at the level of the pulmonary bifurcation, and calculated PWV between ascending and descending portions of the aorta. A flow-compensated acquisition was subtracted from a flow-sensitive acquisition in each TR, giving 10 ms temporal resolution. Gaddum et al. [34] used a similar method, with two velocity-sensitive acquisitions to assess the beat-to-beat variation of PWV and variation during breath-hold maneuvers. Sliding window subtraction of acquisitions from two different gradient waveforms was used to derive a velocity sensitive signal.

**Fig. 7:**
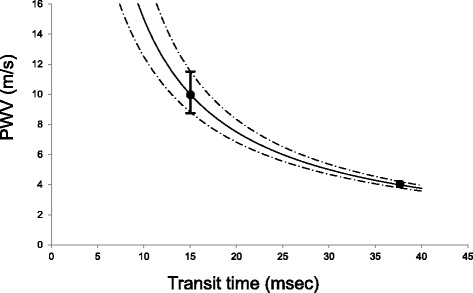
Effect of transit time error on PWV estimation, for an error in transit time of 2 ms and a slice spacing of 150 mm. *Points* show relative errors at 10 and 4 m/s PWV

PWV transit time can be estimated using a variety of methods, including time-to-foot, flow-area and cross-correlation techniques. In studies comparing different methods, transit time using the intersection of the baseline with the upstroke was most reproducible [24, 27] and this was used in this study. However, errors in the determination of transit time non-linearly affect errors in PWV. To illustrate this concept, Fig. 7 shows the estimated error in the PWV measurement, given a 2 ms error in pulse wave transit time (the average variation in transit time in our phantom experiments) and a 150 mm distance between slices. It can be seen that the error increases nonlinearly with decreasing transit times, resulting in wide variation in PWV above 10 m/s. This may explain why the difference between PWV in the phantom experiments was larger for the stiffer butyl rubber phantom than for the latex phantom, and why the scatter increases with PWV in Fig. 6. Similar arguments can be made for the effect of slice spacing at a given PWV, since transit time is linear with slice spacing.

CMR with supine exercise on an ergometer is an alternative method to standard treadmill tests using the Bruce protocol [35]. Supine exercise has previously been used to evaluate strain and transvalvular blood flow [36], aortic compliance [15], diastolic function in type I diabetes [37], and caval flow in Fontan repair [38]. An advantage of treadmill tests is that a higher level of exercise is achievable, however one limitation is that a significant time must elapse between exercise and image acquisition, during which the heart rate can decline rapidly (Fig. 2). Our protocol enables a small number of short duration breath-holds, obtained immediately after cessation of exercise, as well as repeated exercise periods with interspersed imaging.

The PWV results at rest in asymptomatic volunteers are similar to those obtained in previous studies. Nethononda et al. [39] recently reported a large cohort study using the time-to-foot method, with similar age variation as the current study.

Our study found increased PWV during exercise which concurred with previous studies of exercise and pharmacological stress test in asymptomatic volunteers. In a catheterization study in 13 asymptomatic males, aortic PWV increased during supine cycle exercise, along with increased central pressure, and reduced peripheral resistance [14]. PWV changes during dobutamine stress test were studied by Puntmann et al. [40], who found that PWV increased in men but not women. Steeden et al. [15] also found reduced vascular compliance at exercise using pressure-flow relationships in 20 asymptomatic volunteers.

### Limitations

The limited number of short axis slices used in this study may lead to small regional abnormalities in ventricular wall motion being overlooked. Also, the spatial resolution of the cine images (approximately 2.3 mm) used for ventricular function was reduced to compensate for the need for short breath-holds and high temporal resolution. The methods employed in this study estimate average PWV between two slices, rather than local PWV at a point, and therefore are more suited to examination of systemic rather than local aortic disease.

## Conclusion

To our knowledge, this is the first study to demonstrate a valid method of determining PWV during exercise using CMR. Comparable PWV measurements were obtained from the standard 2D gated phase contrast velocity encoding method in phantoms. Normal volunteers showed an increase in PWV with age, and an increase in PWV with exercise. The results suggest this method would be feasible for evaluation of patients with systemic aortic pathologies.

## Abbreviations

CMR: Cardiovascular magnetic resonance
PWV: Pulse wave velocity
RACE: Real-time acquisition and evaluation
STD: Standard gated phase contrast
VENC: Velocity encoding value
